# Ferroptosis in ocular diseases: mechanisms, crosstalk with other cell death pathways, and therapeutic prospects

**DOI:** 10.3389/fmed.2025.1608975

**Published:** 2025-07-08

**Authors:** Shuai Huang, Yuying Sun, Xinxin Yu, Xuan Ren, Lei Wang, Yan Sun, Aijun Deng

**Affiliations:** Affiliated Hospital of Shandong Second Medical University, Weifang, China

**Keywords:** ferroptosis, iron homeostasis, retinal diseases, ocular pathology, cell death mechanisms

## Abstract

**Background:**

Ocular diseases pose a significant threat to visual health, with ferritin ferroptosis playing a critical role in the pathogenesis of many such conditions. Ferritin accumulation, coupled with ferritin autophagy-mediated release of labile Fe^2+^, triggers iron-dependent lipid peroxidation and ferroptosis. These include disruptions in iron metabolism, oxidative stress imbalances, altered intracellular signaling, and changes to the local microenvironment. Such aberrant ferritin deposits not only compromise the structure and function of ocular cells but also accelerate disease progression. Ferroptosis, a newly recognized form of cell death characterized by iron-dependent lipid peroxidation, differs from traditional cell death mechanisms, including apoptosis.

**Materials and methods:**

This review systematically evaluated the role of ferroptosis in ocular diseases using a predefined search strategy. In brief, PubMed was searched for studies published between 2012 and 2025 using keywords combining ferroptosis, ocular diseases, retinal, corneal etc. After excluding non-ocular studies and duplicates, 188 articles were included following a full-text review.

**Conclusion:**

This review examines the molecular mechanisms underlying ferroptosis and its implications for major ocular diseases. It explores how ferroptosis contributes to disease pathology in retinal diseases, offering novel insights for future therapeutic strategies. The potential for targeting ferroptosis pathways with iron modulators holds promise for advancing clinical treatments in ophthalmology.

## 1 Introduction

Ferroptosis is a form of regulated necrosis characterized by iron-dependent lipid peroxidation, first described by Dixon et al. (1). Unlike apoptosis and necrosis, ferroptosis is not a result of conventional cell signaling but rather a unique cell death pathway that involves iron accumulation and the subsequent generation of reactive oxygen species (ROS). Ferroptosis has been implicated in several pathologies, including neurodegenerative diseases, cancer, and cardiovascular disorders, but its role in ocular diseases has gained increasing attention in recent years (2).

In the context of ocular diseases, iron-dependent lipid peroxidation and ferroptosis have been linked to various retinal pathologies, including age-related macular degeneration (AMD), glaucoma, and diabetic retinopathy. The retina, particularly the macula, is highly susceptible to oxidative stress due to its high metabolic activity and exposure to light (3). Iron accumulation, especially ferritin, exacerbates oxidative damage, leading to retinal cell death and progressive vision loss (4).

Understanding the molecular mechanisms that govern ferroptosis in the retina is crucial for developing targeted therapies to treat or prevent these diseases. This review aims to explore the cellular processes underlying ferroptosis and the therapeutic implications for drug development in treating ocular diseases.

### 1.1 Morphological characteristics of ferroptosis

Recent preliminary studies have shown that ferroptosis is morphologically different from apoptosis, necrosis, and autophagy (Table 1). These forms of cell death are characterized by cytoplasmic and organelle swelling, plasma membrane rupture, or the formation of double-membrane vesicles (5). In contrast, the morphological characteristics of ferroptosis mainly focus on the ultrastructure of mitochondria, including the rupture and blebbing of the cell membrane, mitochondrial shrinkage, reduction or even disappearance of cristae, and an increase in membrane density (6). The morphology of the nucleus is normal, but there is a lack of chromatin condensation. Mitochondria are the center of metabolism and an important source of ROS in most mammalian cells. A series of recent compelling evidence has suggested that mitochondria play a key role in ferroptosis (7). For example, some ferroptosis inhibitors can precisely target mitochondria (8).

**Table 1:** Characteristics of different cell death.

**Cell death**	**Ferroptosis**	**Necroptosis**	**Apoptosis**	**Pyroptosis**	**Autophagy**
Morphological features	Cell membrane rupture, mitochondrial atrophy, decreased or disappeared mitochondrial ridges, normal morphology of cell nucleus	Cell membranes are disrupted, and cells and organelles swell and even disintegrate; Inflammatory cell infiltration and activation; Necrosome formation	Pseudopod retraction, nuclear enrichment, chromosome condensation, nuclear membrane rupture, nuclear DNA breaks, apoptotic body formation	Bubble-like protrusions, cytoplasm flattening strongly interlinked with the bottom, dead cells are cabbage or omelet-like	Absent chromosome aggregation, extensive cytoplasmic vacuolation
Predisposing factors	Inflammatory factors, bacteria	Inflammatory factors, bacteria, virus	Radiation, toxin, hypoxia, endoplasmic reticulum stress	Inflammatory factors, toxins, viruses, immune cells	External pressure, hypoxia, hunger, endoplasmic reticulum stress
Signaling pathway	System Xc-, GSH, GPX4, lipids Ros	Caspase-8, RIPK1, TNFα, RIPK3, MLKL, TLR	CytC-AIF-caspase9-caspase3, caspase6, caspase7, caspase8-caspase3, Bcl-2, TRAIL, FASL.TNFR1/2, AIF, ARPP	Caspase1/GSDM; Caspase4/5/11/GSDM; IL-1β, IL-18	mTOR/beclin-1, ULK1/ATG/LC3/p62
Inflammation level	Induce inflammation	Induce inflammation	Anti-inflammatory effect	Induce inflammation	Rarely induce inflammation
Mutagen	RSL5, DIDS, TMZ, MEII, BSO, FIN5, ML162, Erastin	Emricasan, Z-VAD-FMK, Z-IETD-FMK, SM-164, MX69, BV6, Embelin, AT-406, LCL161, GDC-0152, AZD5582	FASL, DCC, UNC5B, LCL161	ZNO-NP, ivermectin, DAC, LPS, Nigericin Sodium, Raptinal	Rapamycin, EBSS
Suppressant	CPX, DFO, DFP, Fer-1, CDDO, Lip-1, VKH2, ALOX15, DKK1	Necrostatin-1, Necrosulfonamide, MLKL-IN, Nec-1S, RIPK1-IN, GSK872, HS-371	XIAP, ILP-2, NAIP, BIRC6, Z-VAD-FMK, Z-IETD-FMK	Necrosulfonamide, VX-765, Z-WEHD-FMK, BAY11-7082, Disulfiram	Chloroquine, 3-MA, NH4Cl, Bafilomycin

### 1.2 Regulatory mechanisms of ferroptosis

**Figure 1:**
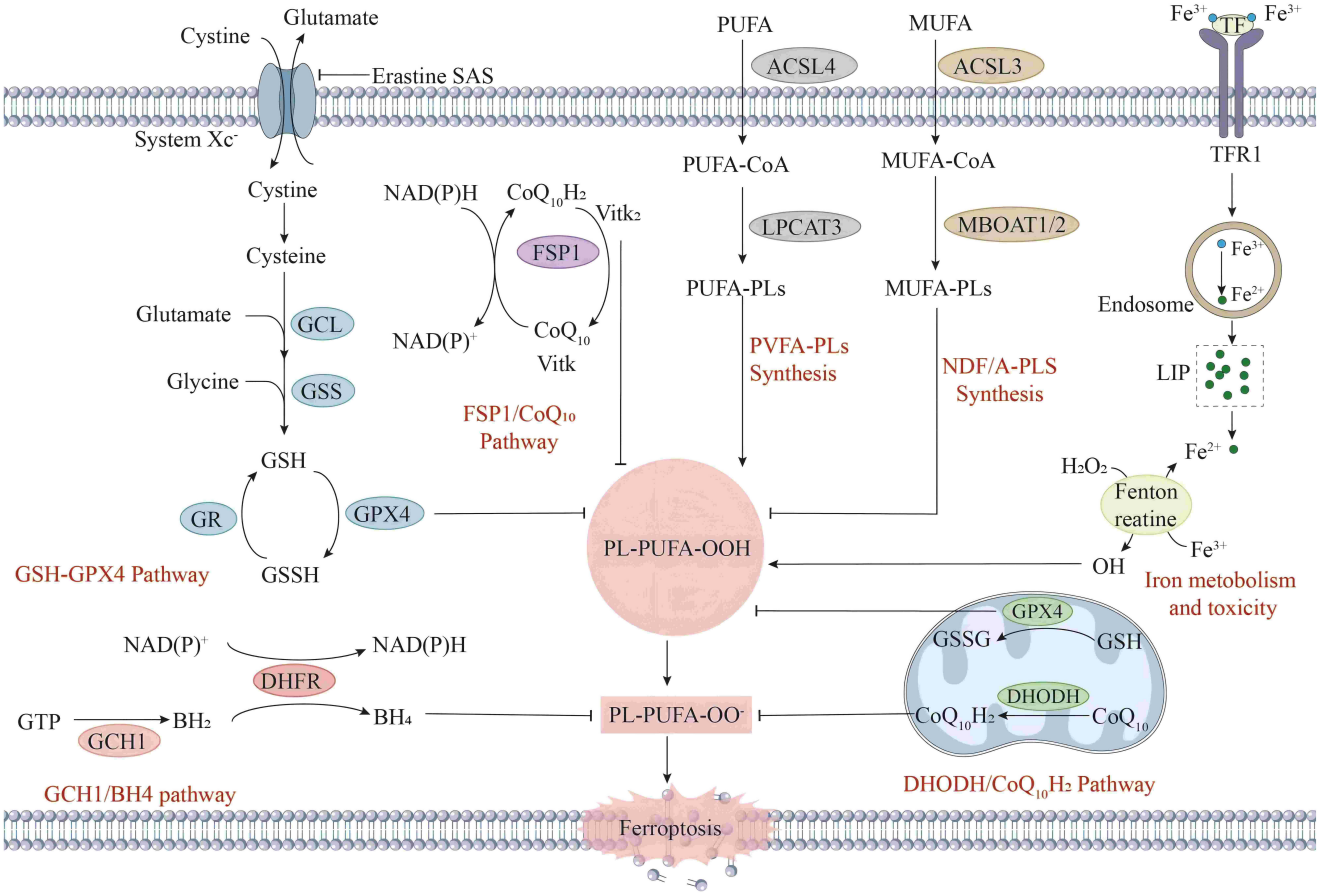
The mechanism of ferroptosis in cells. Ferroptosis is a form of iron-dependent cell death, and its mechanism involves multiple complex biochemical processes. First, in the aspects of iron metabolism and toxicity, extracellular Fe^2+^ enters cells through transferrin receptor 1 (TFR1). After entering the cells, iron ions participate in intracellular redox reactions. Meanwhile, iron ions can be stored in ferritin and released when needed. In the GSH metabolic pathway, glutamate and cysteine are synthesized into γ-glutamylcysteine (γ-Glu-Cys) under the action of γ-glutamylcysteine synthetase (GCL), and then glutathione (GSH) is synthesized under the action of glutathione synthetase (GSS). Under the action of GPX4, glutathione reduces phospholipid hydroperoxides (PL-PUFA-OOH) to phospholipid hydroxides (PL-PUFA-OH), preventing lipid peroxidation. Lipid peroxidation is also a key part of the ferroptosis mechanism. Intracellular polyunsaturated fatty acids (PUFAs) generate phospholipid polyunsaturated fatty acids (PUFA-PLs) under the action of acyl-CoA synthetase long-chain family member 4 (ACSL4) and lysophosphatidylcholine acyltransferase 3 (LPCAT3). In the presence of iron ions, PUFA-PLs are prone to peroxidation reactions, generating phospholipid hydroperoxides (PL-PUFA-OOH). When the activity of GPX4 is inhibited, or the level of GSH decreases, PL-PUFA-OOH cannot be reduced, resulting in the accumulation of lipid peroxidation and eventually triggering ferroptosis. In addition, the GCH1/BH4 pathway and the DHOH/CoQ10H2 pathway are also involved in the intracellular redox balance and antioxidant defense.

The central feature of ferroptosis is redox imbalance (9), and its regulatory network involves multiple metabolic pathways (Figure 1), which show uniqueness in highly metabolic tissues, such as the ocular retina.

Regulatory mechanism (Figure 1): The essence of ferroptosis lies in the depletion of glutathione (GSH) and the decline in the activity of glutathione peroxidase (GPX4), which leads to a reduction in the amount of glutathione reductase catalyzed by GPX4 and excessive accumulation of lipid peroxides (10). Subsequently, Fe^2+^ oxidizes the accumulated lipid peroxides to generate ROS, resulting in an excessive accumulation of ROS and thus facilitating the occurrence of ferroptosis (11). The regulatory mechanisms for ferroptosis mainly focus on aspects such as the glutamate antiporter (System Xc-), GSH metabolism, regulation of the activity of GPX4, and ROS generation (12).

## 2 Mechanisms of ferroptosis in ocular tissues

### 2.1 Dynamic balance of GSH and GPX4

In tissues rich in polyunsaturated fatty acids (PUFAs), such as the retina (13), the GSH-GPX4 axis maintains membrane stability by scavenging phospholipid hydroperoxides (PL-OOH). GPX4 is a phospholipid peroxidation inhibitor, a GSH-dependent enzyme, and an enzyme known to be capable of reducing phospholipid hydroperoxides (14). It can reduce PL-OOH to phospholipid alcohols (PL-OH), inhibiting lipid peroxidation. Under conditions of high glucose or photoinjury, GSH-synthesizing glutamate cysteine ligase (GCL) activity decreases, leading to GSH depletion and GPX4 inactivation, triggering a lipid peroxidation cascade (15). Notably, the expression level of GPX4 in retinal pigment epithelial (RPE) cells was significantly higher than that in other tissues, suggesting a unique defense mechanism against oxidative stress (16).

### 2.2 Regulatory function of System Xc-

System Xc- is a sodium-independent cystine-glutamate antiporter on the cell surface. It is a heterodimeric cell surface amino acid transporter formed by the light chain, the 12-transmembrane transporter solute carrier family 7 member 11 (SLC7A11) (17), and the heavy chain, the single-transmembrane regulatory protein SLC3A2 (4F2hc), which are connected by a disulfide bond. System Xc- has an important role in ocular tissues: on the one hand, it maintains GSH synthesis by the uptake of cystine, and on the other hand, it prevents excitotoxicity by excretion of glutamate (18). In glaucoma, high IOP downregulates SLC7A11 expression, leading to reduced cystine uptake and glutamate accumulation, which doubly exacerbate ferroptosis in retinal ganglion cells (RGCs) (19).

### 2.3 Mitochondrial drive for lipid peroxidation

Ferroptosis's defining hallmark is iron-dependent lipid peroxidation at the plasma membrane, driven by GPX4 inactivation and glutathione depletion, with mitochondrial changes often observed as secondary features. Mitochondria, as iron-rich organelles and the predominant sites of ROS generation, are increasingly recognized as critical hubs for ferroptotic execution (20). Dysregulation of mitochondrial iron homeostasis—a process intricately linked to retinal energy metabolism—exacerbates redox imbalance, driving phospholipid peroxidation and subsequent ferroptotic cell death (21). In photoreceptor cells, mitochondrial damage promotes iron overload and lipid peroxidation (22). Retina-rich docosahexaenoic acid (DHA) is susceptible to oxidation to form toxic aldehydes that exacerbate mitochondrial membrane damage through acyl-CoA synthetase long-chain family member 4 (ACSL4)-mediated phospholipid remodeling (23).

### 2.4 FSP1-CoQ10-NADH: the antioxidant defense line of ubiquinone reduction

Ferroptosis suppressor protein 1 (FSP1) is the first glutathione-independent ferroptosis suppressor. It generates ubiquinol by reducing coenzyme Q10 (CoQ_10_) via NAD(P)H, scavenges lipid peroxyl radicals, and inhibits phospholipid peroxidation (24). This pathway is independent of GPX4 and GSH and can still function when GPX4 is inactivated or GSH is depleted (25). In a sodium iodate (SIO)-induced RPE cell model, oxidative stress suppresses the expression of FSP1 while depleting intracellular reduced CoQ_10_ and reduced nicotinamide adenine dinucleotide (NADH), ultimately triggering lipid peroxidation cascades and leading to RPE cell death. Further studies demonstrate that when GSH is depleted, FSP1 overexpression still exerts cytoprotective effects through a mechanism dependent on the CoQ_10_/NADH cycle, thereby counteracting oxidative stress-induced cellular damage (26).

### 2.5 The GCH1-BH4 pathway: antioxidant regulation by tetrahydrobiopterin

Guanosine triphosphate cyclohydrolase 1 (GCH1) is the rate-limiting enzyme in the biosynthesis of tetrahydrobiopterin (BH4). As a cofactor for nitric oxide synthase (NOS), BH4 inhibits NOS overactivation, thereby mitigating oxidative stress (27). Genome-wide activation screening identified that GCH1 and its metabolites BH_4_/BH_2_ (dihydrobiopterin) inhibit ferroptosis via lipid remodeling, specifically by selectively protecting phospholipids containing two polyunsaturated fatty acyl chains and enhancing the reduced state of CoQ_10_ (28).

## 3 Ferroptosis and ophthalmic diseases

### 3.1 Advances in ferroptosis and related ocular diseases

**Figure 2:**
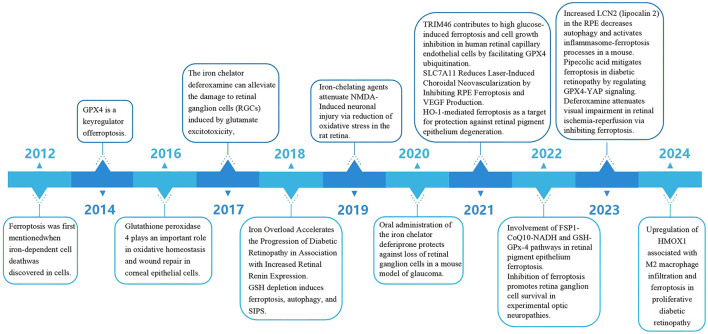
Up to now, the important research progress related to ferroptosis and ophthalmic diseases.

In previous studies (Figure 2), experts mostly associated ferroptosis with tumors. However, as the research on ferroptosis has been getting deeper and deeper, ophthalmologists have also begun to pay attention to the relationship between ferroptosis and ophthalmic diseases. In 2012 (1), the concept of ferroptosis was put forward for the first time. In 2014 (29), GPX4 was identified as a key regulator of ferroptosis. In 2016 (30), it was discovered that GPX4 plays an important role in the oxidative homeostasis and wound repair of corneal epithelial cells, thus linking ferroptosis to ophthalmic diseases. Since then, there have been an increasing number of important studies on ferroptosis related to ophthalmology. *In vitro* experiments mainly focus on RGCs, retinal capillary endothelial cells, and RPE, while *in vivo* experiments mainly concentrate on diseases such as glaucoma and diabetic retinopathy. In 2017 (31), 2019 (32), 2020 (33), and 2022 (34), it was found that inhibiting ferroptosis can alleviate the damage to retinal ganglion cells. In 2018 (35, 36), it was pointed out that iron overload can accelerate the progression of diabetic retinopathy, and at the same time, glutathione depletion can induce iron concentration, autophagy, and stress-induced premature senescence (SIPS). In 2021, it was found that tripartite motif-containing protein 46 (TRIM46) contributes to high-glucose-induced ferroptosis of human retinal capillary endothelial cells by promoting the ubiquitination of GPX4, that SLC7A11 can reduce laser-induced choroidal neovascularization by inhibiting RPE ferroptosis, and that heme oxygenase-1 (HO-1)-mediated ferroptosis can serve as a target for protecting retinal pigment epithelial degeneration. In 2022 (26), studies found that the FSP1 - CoQ10 - NADH and GSH - GPX - 4 pathways are involved in the ferroptosis of the retinal pigment epithelium. In 2023 (37), in a mouse model of dry age-related macular degeneration (dry AMD), the increase in lipocalin 2 (LCN2) in RPE can activate the inflammasome ferroptosis process. Pipecolic acid alleviates ferroptosis in diabetic retinopathy by regulating the GPX4-Yes-associated protein (YAP) signaling pathway. Deferoxamine alleviates retinal ischemia-reperfusion injury by inhibiting ferroptosis. In 2024 (38), in proliferative diabetic retinopathy, the upregulation of heme oxygenase 1 (HMOX1) is related to M2 macrophage infiltration and ferroptosis.

### 3.2 Retinal structure

The embryonic origin of the retina is the optic cup formed by the invagination of the optic vesicle (39). The outer layer of the optic cup forms the RPE, and the inner layer evolves into the retinal neuroepithelium layer (RNL), composed of multiple layers of cells. The main photoreceptive function of the retina is completed within the RNL. The cone cells and rod cells of the photoreceptors (PRs) convert the received light stimuli into nerve impulses, which are transmitted through synaptic connections to bipolar cells and horizontal cells and then to RGCs (40). Then, they are transmitted through the nerve fiber layer and the optic nerve, formed by the axons of ganglion cells, to the lateral geniculate body and finally reach the occipital visual cortex, where visual images are processed and interpreted by the brain to form vision (41).

In addition, the transparency of the retina depends on the tight junctions between the RPE, which form the blood–retinal barrier (BRB) (42). The outer BRB is formed by the close contact between RPE cells and choroidal capillaries and is one of the factors that prevent abnormal components in the choroid from entering the retina (43). The inner BRB is formed by the tight junctions among the endothelial cells of retinal capillaries, pericytes, astrocytes, Müller glial cells (MGCs), and microglia, which is the main factor that prevents blood components from freely entering the retina (44).

### 3.3 The role of iron metabolism in the retina

**Figure 3:**
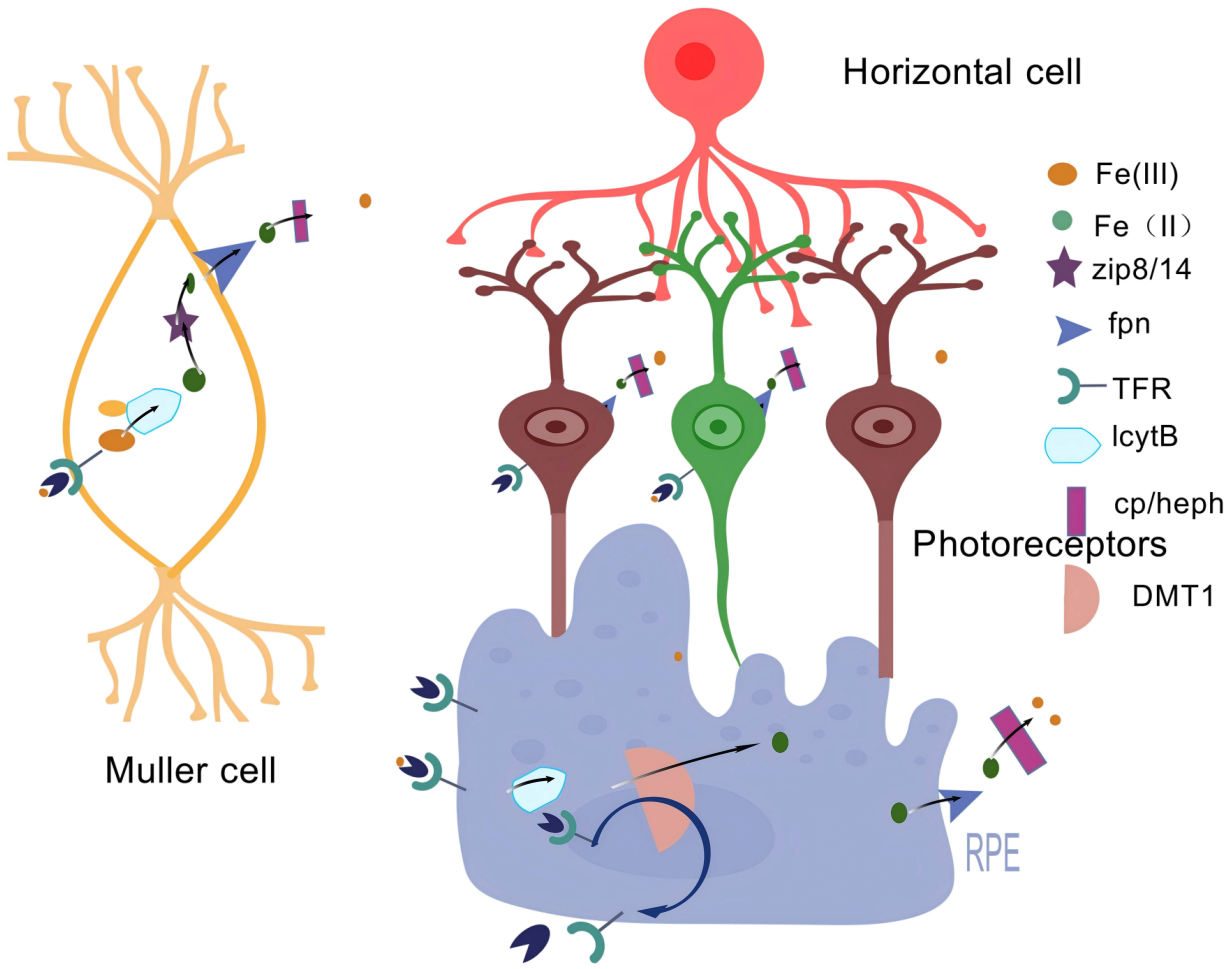
Iron metabolism in the retina. An illustration of iron metabolism in the retina: Free Fe^3+^ binds to transferrin (TF), transported by the TF receptor TFR to cross the BRB. The formed Fe^3+^-Tf-TfR complex translocates intracellularly by cytotoxicity and is reduced to Fe^2+^ by the intracellular ferric reductase LcytB to regulate various chemical reactions. Fe^2+^ is transferred by the divalent metal transporter protein-1 (DMT1). It then translocates to the cytoplasm and transports unutilized or stored Fe^2+^ to the extracellular compartment via Fpn, where it is oxidized by multicopper oxidases such as Cp/Heph to Fe^3+^. Created with BioGDP.com (183).

Iron is one of the metals distributed in the retina (45). Under normal physiological conditions, most of the iron exists in hemoglobin, and other Fe^3+^ combines with transferrin (TF) to form transferrin-bound iron (TBI) (46). Transferrin can protect retinal cells from the potential toxicity of unbound iron and also has a trophic function for the RPE (47). The Fe-S structural motif formed with the participation of iron, whose Fe-S cluster can act as a biosensor and participate in the synthesis of heme (48), is particularly important in retinal pathophysiology (Figure 3). However, when there is an excessive amount of iron in the retina, it will lead to the excessive generation of lipid ROS and an increase in superoxide radicals, which will induce the death of RGCs (49). Meanwhile, it will also activate inflammasomes to promote the release of inflammatory factors, resulting in the death of retinal cells (50).

## 4 Ferroptosis in ocular surface disease

### 4.1 Ferroptosis and corneal injury

The cornea, a transparent avascular tissue at the ocular anterior segment, relies on tight epithelial junctions and redox homeostasis to maintain its refractive and protective functions (51). Corneal injury refers to a state in which the structural integrity of the cornea is damaged due to various external forces, chemical substances, infections, and other factors, resulting in a partial or complete loss of its barrier protection function (52).

Corneal alkali burns, characterized by saponification of cell membranes and limbal stem cell damage, represent a severe form of injury that disrupts iron metabolism and triggers ferroptosis in corneal epithelial cells (CECs) (53). During alkali injury, the corneal oxidative stress response is significantly enhanced, lipid peroxidation occurs, a large amount of intracellular iron ions accumulate, and the activity of GPX4 decreases (54). Wang et al. (55) found that in a mouse model of corneal alkali injury, the expression of ROS was upregulated, oxidative stress increased, the expression of the gene Ptgs2, a key marker of ferroptosis, was elevated, the level of the lipid peroxidation product 4-hydroxynonenal (4-HNE) increased, and the level of the ferroptosis regulator GPX4 decreased, with mitochondrial damage. Nanoparticle-based delivery systems have emerged as promising tools to target corneal ferroptosis. For instance, vascular endothelial growth factor small interfering RNA-TMC-INS-liposome (siVEGF-TIL) not only inhibits neovascularization via VEGF knockdown but also restores System Xc^−^ activity and GPX4 expression, thereby reducing glutamate-induced oxidative stress (56).

Nishant et al. (57) found that mustard gas exposure triggered ROS overproduction, upregulation of the ferroptosis gene ACSL4 promoted lipid peroxidation, and downregulation of SLC7A11 and GPX4 weakened cellular defense. Meanwhile, the phosphorylation level of p38 MAPK was significantly elevated, and its ferroptosis inhibitors, SB202190, reduced ROS production, restored the expression of ferroptosis**-**related genes, and promoted cell survival by blocking this pathway.

These studies have provided abundant theoretical basis and empirical support for creating new methods to treat corneal injuries. They have delved deeply into the mechanism of ferroptosis in corneal injuries and revealed the laws of the occurrence and development of the disease at the cellular and molecular levels, providing crucial clues for the development of targeted treatment strategies.

### 4.2 Ferroptosis in infectious keratitis

Infectious keratitis, a sight-threatening condition often caused by bacterial or fungal pathogens (58), involves complex interactions between microbial virulence factors and host immune responses (59). Recent studies have synthesized bioinformatics analyses to hypothesize and validate that hub genes and pathways associated with ferroptosis play an important role in infectious keratitis (60).

Chen et al. (61) found in bacterial keratitis that pathogens hijack host iron ions by secreting iron carriers, triggering a Fenton reaction-mediated ROS burst and mitochondrial dysfunction. This process is accompanied by the activation of NOD-like receptor thermal protein domain-associated protein 3(NLRP3) inflammatory vesicles and the formation of neutrophil extracellular trapping networks (NETs), which exacerbate corneal epithelial damage and persistent microbial infection. Combined treatment with the ferroptosis inhibitor Fer-1 and levofloxacin significantly reduces inflammatory cytokines, corneal scarring, and Fe^3+^ accumulation while upregulating the expression of GPX4 and SLC7A11. Inhibiting ferroptosis holds promise as a novel therapeutic strategy to mitigate inflammation and scarring in BK.

In this study (62), we designed an iron-based metal-organic framework (MIL-101) loaded with riboflavin nanoparticles for the treatment of bacterial keratitis through the photothermal synergistic dual mechanism of iron-death and immunomodulation: MIL-101 produces a mild thermal effect and releases Fe^3+^, which induces the ROS flare and bacterial lipid peroxidation through the Fenton reaction, triggering an iron-death-like bacterial effect. At the same time, the continuous release of riboflavin can inhibit the activation of macrophage nuclear factor kappa-B (NF-κB)pathway, reduce the secretion of pro-inflammatory factors, and alleviate the excessive inflammatory response. Combined with the trans-epithelial and painless delivery of SilMA hydrogel microneedle patch, it breaks through the corneal barrier and prolongs the retention time of the drug, realizing the synergistic effect of high bactericidal efficacy and immunomodulation.

### 4.3 Ferroptosis and dry eye disease

Dry eye syndrome, also known as keratoconjunctivitis sicca, is a common ocular surface disease caused by multiple factors, including insufficient tear secretion, excessive evaporation, and abnormal tear composition (63).

**Figure 4:**
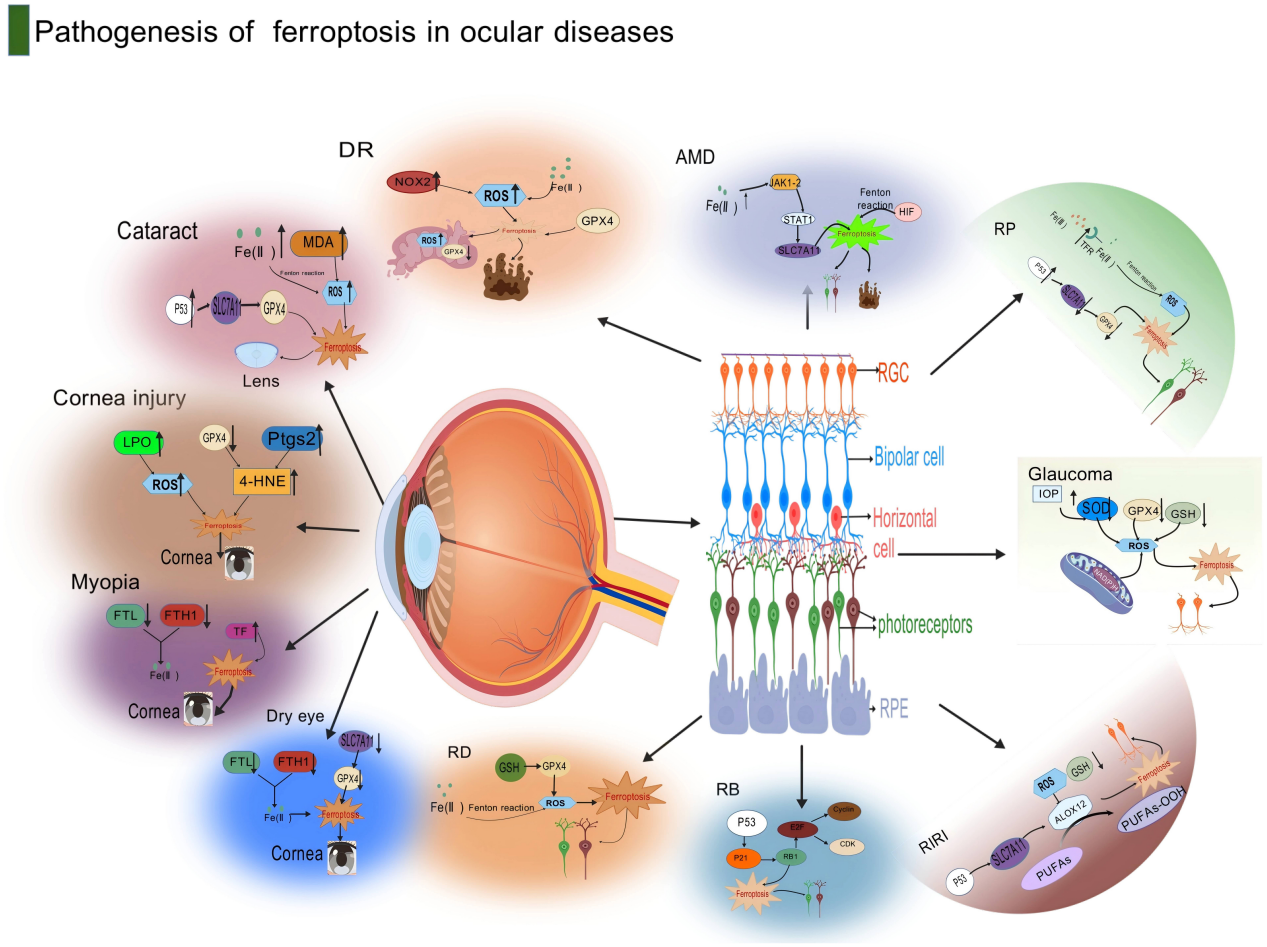
This diagram illustrates the pathogenesis of ferroptosis in ocular diseases. Centered around the anatomical structure of the eye, the diagram lists in detail the molecular mechanisms related to ferroptosis in various eye diseases. In cataracts, pathways such as LPO, ROS, Pitx3, and 4-HNE are involved; corneal injury is associated with Myopia, FTL, and TF; dry eye is related to FTH1 and TF. Diabetic retinopathy in retinopathy is related to RDS, MDA, and SOD2; age-related macular degeneration involves RPE, RGC, and Bruch's membrane; glaucoma is associated with RGC, IOP, and RPE. The diagram also marks several key molecules and cellular structures related to ferroptosis, such as retinal pigment epithelium (RPE), retinal ganglion cells (RGC), bipolar cells (Bipolar cell), and photoreceptors (Photoreceptors), indicating that ferroptosis plays an important role in the pathogenesis of ocular diseases through these molecular and cellular mechanisms. Created with BioGDP.com (183).

Previous studies have shown that mechanisms such as necrosis, apoptosis, and pyroptosis are involved in the progression of dry eye syndrome (DES) (64). In addition, studies have already confirmed that ferroptosis has a good protective effect on corneal epithelial defects (63). This has triggered the scientific community to conduct in-depth explorations on whether ferroptosis is involved in the pathogenesis of DES. Zuo et al. (65) found through single-cell transcriptomics sequencing research that ferroptosis-related pathways were enriched in human corneal epithelial cells (HCEC) treated with hyperosmotic solution, and there were changes in the mRNA expression of key genes. For example, the level of GPX4 decreased while the levels of transferrin receptor (TFRC) and ACSL4 increased. There were also changes in protein expression levels. For instance, the expression level of the ACSL4 protein decreased, and the contents of the ferritin subunit (FTL) and ferritin heavy chain (FTH1) decreased (Figure 4).

In a mouse dry eye model induced by scopolamine hydrobromide, an increase in the level of malondialdehyde (MDA) and downregulation of the protein expressions of SLC7A11 and GPX4 were detected. Astaxanthin (AST) can significantly improve its symptoms, protect the mitochondrial structure, inhibit ferroptosis, and activate autophagy, thus effectively protecting corneal epithelial cells from ferroptosis damage related to dry eye syndrome both *in vitro* and *in vivo* (66). Heme oxygenase-1 (HMOX1) is a key regulatory gene of ferroptosis in atherosclerosis (67). An increase in the expressions of HMOX1 and hypoxia-inducible factor-1α (HIF-1α) was detected in the corneas of a rat DES model. Qingxuan Runmu yin (QXRMY) effectively reduced their expressions in the corneas, repaired corneal damage, protected conjunctival goblet cells, reversed the oxidative stress induced by DES, and slowed down the progression of DES (68).

The above scientific research results have demonstrated that ferroptosis plays an important role in the pathogenesis of DES and is highly likely to become a new target for the treatment of DES.

## 5 Ferroptosis in anterior segment disease

### 5.1 Ferroptosis and cataract

Cataracts are the leading cause of blindness worldwide and are driven primarily by oxidative stress and protein aggregation, leading to progressive lens opacities (69). In the early stage of the disease, the clinical manifestations of cataracts are often not significant. However, as the disease progresses, patients will gradually experience symptoms such as decreased vision, blurred vision, glare, strabismus, diplopia, and reduced contrast sensitivity, which seriously interferes with patient's daily activities and has a great impact on their quality of life (70).

Recent evidence suggests that ferroptosis is a key pathologic mechanism in cataracts. In UV-exposed lens epithelial cells (LECs) (71), iron mutations are characterized by mitochondrial cristae disassembly, iron overload, and downregulation of GPX4 and SLC7A11. This leads to GSH depletion and lipid peroxidation manifested by elevated MDA and 4-HNE adducts (72). Using bioinformatics methods, ferroptosis-related DEGs such as peroxisome proliferator-activated receptor gamma (PPARG) and Silent information regulator 1(SIRT1), as well as hub genes, were identified in the GSE3040 database, and their expressions were found to be elevated in the cataract cell model (73).

Sun et al. (74) found higher expression levels of miR-224-3p, TFR1, and IL-6 and lower levels of ACSL4 and GPX4 in LECs from patients with high myopic cataracts (HMC). A significant reduction of mitochondria was observed under electron microscopy, and miR-224-3p was demonstrated to enhance the viability of HLECs by targeting the regulation of ferroptosis and inflammation through ACSL4.

Diabetic cataract: hyperglycemia-induced epigenetic modifications (hypermethylation of PRNP and SLC39A8) lead to Fe ion imbalance with disturbed mitochondrial dynamics, enhancing oxidative stress in DC (75). Mendelian analysis identified four core genes—Hspa5/Nfe2l2/Atf3/Stat3—that are associated with oxidative stress and ferroptosis in DC. Genetic modeling demonstrated functional interactions between oxidative stress and ferroptosis genes in DC, suggesting that they may act as “bridges” in the pathogenesis of DC (76).

Inhibition of ferroptosis shows promise as a therapeutic target for cataracts. Mi et al. (77) found that melatonin (MT) restores redox balance by activating SIRT6, which in turn upregulates GPX4 and inhibits NCOA4-mediated ferroptosis, ultimately delaying cataract progression. Ni et al. (78) showed that upregulation of Notch signaling alleviated ferroptosis by decreasing the levels of FTH1 and p53 and enhanced the expression of Nuclear factor-erythroid 2 related factor 2(Nrf2), GPX4, and SLC7A11 in an *in vitro* LEC assay. In addition, downregulation of Notch signaling promotes ferroptosis in LEC by impairing the Nrf2/GPX4 antioxidant pathway, which contributes to the development of cataracts. The Notch signaling pathway is emphasized as a potential therapeutic target to prevent or attenuate ARC progression.

## 6 Ferroptosis in orbital disease

### 6.1 Ferroptosis and Graves' ophthalmopathy

Graves' ophthalmopathy, also known as thyroid-associated ophthalmopathy (TAO) or Graves' ophthalmopathy (GO), is an organ-specific autoimmune disease involving orbital tissues (79). Its incidence ranks first among orbital diseases, and it occurs in 25–40% of patients with Graves' hyperthyroidism (80).

Ferroptosis plays a very important role in the pathogenesis of GO (81). A recent study has shown that enhanced glycolysis can enhance the resistance of orbital fibroblasts (OFs) in GO patients to ferroptosis, thereby resisting tissue fibrosis (82). Wang et al. (83) observed inhibition of lipogenesis in GO-OFs after simvastatin administration, along with a series of ferroptosis features, such as altered mitochondrial structure, increased levels of ROS, intensified lipid peroxidation, and an increase in both mRNA and protein expression of ACSL4. This suggests that simvastatin can inhibit the growth of GO-OFs by inducing ferroptosis.

Ye et al. (84) used bioinformatics methods to perform an intersection operation on ferroptosis-related genes from the human source and the FerrDb V2 database, as well as the differential genes of GO, and successfully screened out 11 ferroptosis-related differential genes (F-DEGs). Li et al. (85) identified and validated two iron-dead OFGs with diagnostic and therapeutic potential in TAO orbital adipose tissue, suggesting that the downregulation of ACO1 and HCAR1 may be potential molecular targets in the pathogenesis of TAO.

## 7 Ferroptosis in fundus disease

### 7.1 Ferroptosis and diabetic retinopathy (DR)

DR is a chronic, progressive, and severely vision-threatening retinal microvascular disease associated with persistent hyperglycemia and other diabetes-related conditions such as hypertension. It seriously affects the quality of life of middle-aged and elderly people (86). Is characterized by BRB breakdown (42), retinal neovascularization (87), and neurodegeneration (88). As a common and specific complication of diabetes, ~50% of diabetic patients may develop this lesion 10 years after the onset of diabetes, and the proportion reaches 80% after more than 15 years (89). The pathological grading of DR includes two types: proliferative diabetic retinopathy (PDR) and non-proliferative diabetic retinopathy (NPDR) (90).

Recent studies have found that oxidative stress plays a key role in it (91). The chronic increase in blood glucose levels can lead to metabolic disorders in retinal endothelial cells (EC), trigger oxidative stress responses, promote apoptosis of these cells, increase vascular permeability, and then result in the destruction of the BRB (92). Some blood components, inflammatory mediators, and lipid peroxides enter the retina and cause macular edema (93). In human retinal capillary endothelial cells (HRCECs) exposed to HG, TRIM46 induces ferroptosis by promoting GPX4 ubiquitination and degradation, leading to GSH depletion and lipid peroxidation. The ferroptosis inhibitor Fer-1 partially restores cell viability after HG treatment, suggesting that the TRIM46-GPX4 pathway serves as a key regulatory axis for ferroptosis in DR (94). In addition, the high glucose environment also significantly increased ROS and LPO levels, which inhibited GPX4 activity leading to disruption of mitochondrial integrity (95).

Iron overload axis: HG disrupts iron homeostasis in RPE cells through TXNIP-mediated ferritinophagy, releasing free iron that drives Fenton reaction-dependent ROS production (96).

Neurodegeneration: Müller cells in the DR also exhibited ferroptosis features, including downregulation of SLC7A11, GSH, and GPX4 and upregulation of ACSL4 and MDA, leading to glutamate excitotoxicity (97). Treatment with resveratrol (RSV) suppressed changes in neurologic function and mitochondrial morphological alterations in their DR retinas (98).

Vascular dysfunction: Characteristic changes in ferroptosis-related markers, including GPX4, FTH1, ACLS4, TFR1, and cyclooxygenase-2(COX-2), were detected in the retinal fibrovascular membranes of patients with proliferative diabetic retinopathy, cultured Human Retinal Microvascular Endothelial Cells (HRMECs), and the retinas of diabetic mice (99).

Sixty-three ferroptosis-associated differentially expressed marker genes (FA-DEMGs) were identified by single-cell transcriptome sequencing, which was significantly enriched in peroxiredoxin activity, ferroptosis, mitochondrial autophagy, and autophagy, contributing to future advances in DR therapy (100). Exploring the interplay between ferroptosis and autophagy in Müller cells may uncover novel therapeutic windows. Additionally, developing intravitreal nanocarriers loaded with ferroptosis inhibitors could provide sustained retinal drug delivery, addressing the chronic nature of DR (101).

### 7.2 Ferroptosis and AMD

AMD, a leading cause of irreversible blindness in the elderly (102), involves progressive RPE dysfunction and choroidal neovascularization (CNV) (103). Ferroptosis is a critical driver of RPE cell death in AMD, distinct from the apoptotic pathways observed in other ocular pathologies (104).

In laser-induced CNV models, ferroptotic RPE cells exhibit mitochondrial cristae disassembly, iron overload, and upregulation of GPX4 ubiquitination markers. This is accompanied by lipid peroxidation products, including 4-hydroxynonenal, and inflammatory mediator release, exacerbating subretinal inflammation (105).

Oxidative stress-hypoxia crosstalk: Oxidative stress agents, such as sodium iodate (SI)-treated RPE cells under hypoxia, show augmented ferroptosis via hypoxia-inducible factor (HIF)-mediated Fenton reaction activation, distinct from normoxic conditions (106).

Immune-mediated iron dysregulation: Interferon-γ (IFN-γ) downregulates SLC7A11 via the JAK1-2/STAT1 axis, leading to GSH depletion, triggering ferroptosis in RPE cells (107).

Epigenetic regulation: In transcriptomics, Wu and Sun first completed the construction of the circRNA-miRNA-mRNA network in AMD using 23 circRNAs, 8 miRNAs, and 14 ferroptosis-related genes in gene sequencing (108). Using bioinformatics techniques, co-expression networks of differential genes between AMD subtypes were constructed, revealing the regulatory role of ferroptosis hub genes at the level of transcription factors and miRNAs (109).

Targeting the specific mechanism of action of ferroptosis in AMD, Huang et al. (110) developed a melanin nanomedicine ConA-MelNPs with chelating iron ion properties that protect against RPE cell damage and attenuate severe mitochondrial damage induced by oxidative stress, thereby restoring retinal homeostasis. Zhu and Yu (111) demonstrated that ferrous ammonium citrate induced ferroptosis in retinal RPE cells in a dry AMD model. Further studies revealed that salidroside, an antioxidant with antioxidant properties, significantly attenuated oxidative stress-mediated retinal damage by activating the nuclear factor Nrf2/SLC7A11/GPX4 signaling axis.

All of the above studies can confirm that ferroptosis plays a key role in the pathophysiology of AMD. Therefore, it would be a new treatment approach to increase the exploration of the clinical application of ferroptosis inhibitors in AMD.

### 7.3 Ferroptosis and retinitis pigmentosa (RP)

Retinitis pigmentosa (RP) is a hereditary eye disease, mainly with autosomal recessive inheritance, accompanied by dystrophic degenerative changes (112). Patients initially show a decline in night vision. As they grow older, there is a progressive decline in central vision, visual field defects, and color vision loss, which seriously impairs the quality of life of the patients (113). Its pathological factors are mainly related to the death of photoreceptor cells, cone cells, and rod cells, as well as the degeneration of RPE cells (114).

Recently, more and more studies have found that there is also the phenomenon of abnormal iron homeostasis in the pathophysiology of RP. When two iron chelators, VK28 and VAR10303, were applied to the rd10 mouse model, it could be observed that the survival rate of photoreceptor cells, such as rod cells, was significantly improved, which greatly improved the visual function of the mice (115). Injecting Fe^2+^ into the vitreous cavity of mice can cause lipid peroxidation, a decrease in the expression level of GPX4, retinal oxidative damage, and pan-retinal fluorescence. Further studies have confirmed that cone cells are more vulnerable to the influence of Fe^2+^ and die faster than rod cells (116).

In the RP model established using H_2_O_2_ to induce the cone cell line 661W cells, it was found that there were excessive accumulations of MDA and increased consumptions of SOD and GSH (117). All these characteristics are closely related to ferroptosis. Furthermore, it was proposed that the p53 gene might be the key gene for regulating RP. Studies have shown that when p53 increases, the expressions of SLC7A11 and GPX4 will decrease (118). Iron inhibitors can reduce the production of ROS to influence the occurrence of the p53/SLC7A11 signal transduction axis during the progression of RP. Moreover, when FSE, the extract of Lycium barbarum and Salvia miltiorrhiza, was applied to the RP mouse model, it could improve the death of photoreceptor cells (119).

Xiong et al. applied a traditional Chinese medicine combination named QST, which is used for treating ophthalmic diseases, to the RP mouse model. Compared with the control group, the retinal vascular structures of the mice taking QST were clearer, the blood flow volume increased, the intracellular iron level decreased, and the contents of SOD and GSH increased. Further studies have confirmed that QST inhibits ferroptosis by influencing the NRF2/GPX4 signal transduction pathway and thus alleviates RP (120). Through bioinformatics analysis techniques, six central genes related to ferroptosis, namely Jun, Stat3, Hmox1, Atf3, Hspa5, and Ripk1, were also identified in the RP mouse model (121). Since there are currently no effective treatments for RP, the discovery of the ferroptosis mechanism in RP is of great importance. Using some iron chelators to reduce lipid peroxidation and relieve iron overload may become a new idea for the treatment of RP.

### 7.4 Ferroptosis and glaucoma

Glaucoma is a progressive optic neuropathy characterized by RGC death and visual field loss (122). Fluctuations in intraocular pressure (IOP), insufficient blood supply to the optic nerve, malnutrition, trauma, hypoxia-induced inflammatory responses, and degeneration of RGCs are the basic factors for its onset (123), among which the increase in pathological intraocular pressure (ph-IOP) and the death of RGCs are the key factors (124). In glaucomatous retinas, ferroptosis is marked by mitochondrial cristae disassembly (125), iron overload, and downregulation of GPX4 in RGCs.

New Perspectives on ion channels: Liu et al. (126) reversed ferroptosis in human trabecular meshwork cells by knocking down the ion channel protein Piezo1. Mitochondrial damage was reversed, GPX4 levels increased, and lipid peroxidation was reversed. Piezo1 was verified to be a key mediator of ferroptosis in TMC. Zhang et al. (127) established that transmembrane and coiled-coil domain protein 1 (TMCO1) induces ferroptosis and extracellular matrix (ECM) deposition in HEMC through the ERK/MAPK pathway. These studies provide new insights into the pathogenic mechanisms of glaucoma.

Oxidative stress: Wang et al. (128) found that silicone oil (SiO) inhibits the proliferation of HTMCs by upregulating fibrosis-related markers and elevating ferroptosis levels through the ROS/NOX4/Smad3 signaling axis. Gene knockdown experiments further revealed that SiO-induced ferroptosis and ROS elevation are mediated by NOX4 upregulation and Smad3 activation, highlighting the critical role of ferroptosis and this signaling axis in SiO-induced intraocular pressure elevation. These findings identify potential therapeutic targets for mitigating postoperative complications associated with SiO tamponade. Insights gained from this study identify potential therapeutic targets to alleviate the postoperative complications associated with SiO filling in ophthalmic surgery.

Many experts are working on novel therapeutic strategies to address the mechanism of ferroptosis in glaucoma. It has been experimentally verified that oral administration of deferiprone (DFP) can chelate excessive Fe2^+^, alleviate oxidative stress, and have a protective effect against RGC damage (4). In RGC-5 cells treated with oxygen-glucose deprivation/reoxygenation (OGD/R), there were decreases in cell viability, disorders of iron homeostasis, and enhancements of oxidative stress. It was also confirmed that the overexpression of gamma-glutamyl transpeptidase 1 (GGT1) increased the synthesis of GSH, reduced the accumulation of MDA, and alleviated the damage to RGC cells (129). Zhou et al. (130) discovered that oral administration of 7,8-dihydroxyflavone (7,8-DHF) increases the intestinal microbial metabolite indoleacrylic acid (IDA). IDA protects RGCs by activating the AhR-ALDH1A3-FSP1 pathway, promoting the reduction of CoQ10, reducing lipid peroxidation, and enhancing antioxidant capacity.

We have reasons to suspect the importance of ferroptosis in the early stage of glaucoma onset. Currently, there are no good treatment methods for glaucoma in the early stage. New treatment methods can be sought by further studying the pathogenesis of ferroptosis in glaucoma, such as oral iron chelators and gene-targeted therapies, so as to slow down the progress of glaucoma (131).

### 7.5 Ferroptosis and retinal ischemia-reperfusion injury

Retinal ischemia-reperfusion refers to the restoration of blood perfusion after the interruption of retinal blood flow and the retina is in an ischemic state. It will cause retinal dysfunction and extremely serious damage to the retinal structure, resulting in irreversible visual impairment (132). Its damage mechanism is an important pathological basis for a variety of eye diseases, such as retinal vascular occlusion, glaucoma, retinopathy of prematurity, and diabetic retinopathy (133). Its pathogenesis is related to several theories, including inflammatory responses, free radical generation, intracellular calcium overload, and apoptosis (134). Recent studies highlight ferroptosis as a dominant cell death modality in RIRI, distinct from apoptotic pathways observed in other ocular pathologies (135).

In the rat model of IR injury, within 3 days, it was detected that retinal Fe^2+^ and the oxidative stress markers, ROS and MDA, increased rapidly and reached the maximum peak at 48 h. The levels of proteins related to ferroptosis, such as transferrin receptor (TFR), increased over time, while the protein levels of SLC7A11 and GPX4 decreased. Different degrees of mitochondrial shrinkage were observed in RGCs, photoreceptor cells, and retinal pigment epithelial cells (136).

In a recent study, Mei Tingfang et al. found that lipocalin 2 (LCN2) was upregulated in the retinal IR model. Through a series of protein level tests, it was found that after inducing the IR injury model in LCN2-TG mice, the total GSH content in the retina, the protein level of GPX4, and the light chain FTL decreased, while the level of the oxidative stress product MDA increased. This indicates that the upregulation of LCN2 promotes ferroptosis in IR injury (137).

The p53 gene can participate in ferroptosis through the combined effects of regulating cellular lipid metabolism, ROS accumulation, and intracellular iron ion levels. In the canonical GPX4-dependent pathway, p53 suppresses SLC7A11 to reduce cystine uptake, leading to GSH depletion and impaired GPX4 activity, thereby promoting ferroptosis. In the non-canonical GPX4-independent pathway, p53 inhibits SLC7A11 to release lipoxygenases ALOX12/ALOXE3, which catalyze the peroxidation of membrane-bound PUFAs to initiate the ferroptotic program (138). When using RNA-seq technology based on techniques to observe the signal cascade reactions related to RGC death in retinal IR injury, it was found that the level of the Steap3 gene responsible for encoding the production of Fe^2+^ and Fe^3+^ was more than twice as high as that in the normal group. Its family genes, Steap1 and Steap4 genes, were also upregulated to varying degrees, demonstrating that ferroptosis also plays an important role in IR injury. RGC death involves various types of programmed cell death, such as pyroptosis, necroptosis, and regulated necrosis. Now, ferroptosis is also involved, and the interactions among these pathways jointly affect RGC death after IR injury (139). Therefore, it is a crucial factor in the treatment of retinal ischemia-reperfusion to urgently develop a therapy that can regulate many programmed cell death (PCD) pathways.

### 7.6 Ferroptosis and retinoblastoma

Retinoblastoma (RB), a pediatric intraocular malignancy arising from retinal progenitor cells, is characterized by biallelic inactivation of the RB1 tumor suppressor gene (140). Its initial clinical manifestation is leukocoria, followed by symptoms such as corneal edema, vitreous cavity turbidity, iris neovascularization, congestion and edema in the conjunctiva, and strabismus (141). Currently, the basic treatments include intravenous chemotherapy (IVC), intra-arterial chemotherapy (IAC), intravitreal chemotherapy (IvitC), combined laser and cryotherapy, and enucleation of the eyeball, which is required for advanced cases (142). Since this disease is prone to intracranial or distant metastasis and poses a serious threat to children's lives, researching its pathogenic mechanism and treatment targets has always been a top priority in ophthalmic research.

Carboplatin is globally recognized as the first-choice drug for the treatment of retinoblastoma (143). It can be injected intravenously as a single drug or used in the technique of ophthalmic artery chemosurgery (OAC) (144). Moreover, it can also be used in combination with vincristine and etoposide (145). Since anti-tumor drugs require long-term and continuous treatment, children are prone to acquire multiple drug resistance (MDR). With the deepening of research on ferroptosis in the field of ophthalmology, researchers have established a method to selectively induce ferritin to eliminate MDR RB cells through autophagy. An itaconate derivative, 4-octyl itaconate (4-OI), has been proven to promote ferroptosis by selectively degrading anti-ferritin, GPX4, solute carrier family 40 member A1 (SLC40A1), and organelles under safe and effective conditions, thus eliminating MDR RB cells, which may provide clinical workers with new targets for the treatment of acquired drug resistance (146). YAP is an oncoprotein existing in the cytoplasm. When activated, it can translocate to the nucleus and initiate the transcription of genes that regulate cell division and apoptosis (147). Its expression is decreased in retinoblastoma. The overexpression of YAP may induce ferroptosis by mediating mitochondrial fatty acid β-oxidation and lipid peroxidation, further promoting the apoptosis of RB cells, inhibiting their apoptotic proliferation, and PY-60 can further enhance the pro-apoptotic effect of YAP and increase the sensitivity of RB cells to cisplatin/etoposide (148). Actively exploring the specific mechanism of ferroptosis in retinoblastoma is the focus of the next research step, aiming to improve the treatment effect of retinoblastoma and the survival time of patients.

### 7.7 Ferroptosis and retinal detachment

Retinal detachment (RD) refers to an ophthalmic emergency in which the neurosensory retina is separated from the retinal pigment epithelium (149). The death of photoreceptor cells is the main cause of vision loss in retinal detachment (150). Surgical reattachment of the retina remains the primary treatment method at present, and its prognosis is closely related to the duration and extent of the detachment, as well as the timeliness of the surgery (151).

Oxidative stress is a prominent feature in the eyes of patients with primary rhegmatogenous retinal detachment (RRD) and is directly related to the severity of the detachment (152). It also plays an important role in retinopathy of prematurity (ROP) (153). Treating RPE cells with the iron chelator deferoxamine (DFO) can lead to a reduction in intracellular iron ions (154), an increase in genes related to iron transport, a significant elevation in the level of hypoxia-inducible factor HIF2α, and impairment of mitochondrial function, indicating that the RPE is the main target tissue for DFO toxicity, resulting in atrophy and degeneration of the RPE and posing a risk of retinal detachment. The mechanism is related to the upregulation of HIF2α and mitochondrial function defects (155).

These findings have provided potential targets and strategies for the treatment of retinal detachment. By regulating ferroptosis-related pathways or substances, it is possible to develop more effective treatment methods to slow down or prevent cell damage and vision loss caused by retinal detachment.

## 8 Ferroptosis and other ocular diseases

### 8.1 Ferroptosis and myopia

Myopia refers to a condition in which parallel rays of light focus in front of the retina after passing through the refractive system of the eyeball, resulting in blurred vision. It is a type of refractive error and is currently one of the most common diseases (156). With the increase in the global myopia population, when it progresses to high myopia, it can lead to a series of other blinding eye diseases, such as retinal detachment, choroidal neovascularization, cataract, glaucoma, macular atrophy, and posterior staphyloma (157). Currently, regarding refractive phenotypes, many international research centers have carried out genome-wide association studies (GWAS) and meta-analyses, aiming to deeply explore the potential genetic basis and its mechanism in the occurrence and development of myopia (158).

In this study, through high-throughput TMT labeling and parallel reaction monitoring (PRM) verification technology, the differences in corneal protein expression between patients with high and low degrees of myopia were analyzed in the corneal stromal tissues obtained from myopic patients who underwent small incision lenticule extraction (SMILE) surgery. A total of 2,455 proteins were found in the Homo sapiens Protein Atlas, among which 103 proteins showed expression differences. Through data-independent acquisition (DIA) spectra and functional enrichment analysis, it was revealed that the proteomic characteristics of patients with high and low degrees of myopia were different. In high myopia (patients with spherical equivalent SE > −6.00D), there was disordered iron metabolism, accelerated corneal oxidative stress, decreased expression levels of FTL and FTH1, and increased serum transferrin (TF). It indicates that ferritin may serve as a biomarker and provide new ideas for understanding the pathogenesis of high myopia. However, further basic experiments and clinical studies are still needed to determine its role in the diagnosis and treatment of myopia (159).

## 9 Crosstalk between ferroptosis and other forms of cell death

### 9.1 Ferroptosis and apoptosis

The interaction between ferroptosis and apoptosis is particularly pronounced under oxidative stress conditions and centers on the bidirectional regulation of mitochondrial dysfunction and redox imbalance (160); disruption of mitochondrial morphology, ROS-induced oxidative stress, apoptosis, and ferroptosis observed in selenium-stressed zebrafish embryos; and co-administration of ferroptosis and apoptosis inhibitors ameliorates embryonic eye developmental defects (161). Wang et al. (162)'s use of enhanced nanoparticles combined with the ferroptosis-apoptosis anticancer pathway under the synergistic effect of ultrasound can inhibit the growth and metastasis of *in situ* uveal melanoma. In conclusion, we suspect that ferroptosis and apoptosis may play a key role in some ocular diseases, which provides new diagnostic and therapeutic ideas for the treatment of ophthalmic diseases in future. A study utilizing a mouse retinal ischemia-reperfusion (IR) model and RNA-seq analysis revealed that multiple PCD pathways, including apoptosis, necroptosis, pyroptosis, and ferroptosis, are simultaneously activated in ischemic RGCs. Inhibition of death receptors or reduction of Fe^2+^ significantly improved RGC survival, suggesting that concurrent regulation of multiple PCD pathways represents a critical strategy for treating retinal IR injury (139).

### 9.2 Ferroptosis and pyroptosis

At the core of the interaction between ferroptosis and pyroptosis lies the cascade amplification of oxidative stress and inflammatory responses (163). Galina et al. (164) used RNA-seq data to reveal that necroptosis, pyroptosis, and ferroptosis are collectively involved in the pathologic damage of retinal ischemia-reperfusion. Among them, TNF, glutamate, and Fe^2+^ produced by Steap3 may be the key players in the simultaneous triggering of ischemic RGC. We hope to propose a dual strategy of “iron metabolism regulation and inflammation inhibition”, breaking through the traditional single-target limitations. In the oxygen-glucose deprivation/reperfusion (OGD/R)-induced RPE injury model, excessive ROS generation triggers iron-dependent lipid peroxidation, leading to ferroptosis, while simultaneously activating the NLRP3 inflammasome. This activation mediates Gasdermin D (GSDMD) cleavage via caspase-1 and releases IL-1β and IL-18, inducing pyroptosis. Inhibition of phospholipase D 1/2 (PLD1/2) blocks ROS accumulation, iron metabolic disorders, and NLRP3 activation simultaneously, indicating that both processes share upstream regulatory nodes controlled by the PLD signaling pathway. These findings suggest that targeting PLD could be a key strategy to disrupt the crosstalk between ferroptosis and pyroptosis (165).

### 9.3 Ferroptosis and autophagy

The interaction between ferroptosis and autophagy centers on the synergistic regulation of iron homeostatic imbalance and iron autophagy (166). Liu et al. (167) found that vitreous humor secretes Glia maturation factor-β (GMFB) in a high-glucose environment, which in turn translocates the ATPase H^+^ Transporting V1 Subunit A(ATP6V1A) from lysosomes, alkalizes lysosomes in RPE cells, and the ACSL4 protein ends up being digested in lysosomes, where an abnormal autophagy-lysosomal degradation process leads to the production of lipid species and ultimately induces ferroptosis in RPE. We hope to develop an autophagy-ferroptosis interaction-based scoring system to predict disease progression. In corneal epithelial cell injury of dry eye disease, impaired autophagic flux leads to accumulation of sequestosome 1 (SQSTM1), which upregulates ACSL4 to promote the insertion of iron-dependent lipid peroxidation. Concurrently, dysregulated ferritinophagy caused by autophagic abnormalities results in free Fe^2+^ accumulation, further driving Fenton reactions and ROS production. Inhibition of SQSTM1 or ACSL4 disrupts this crosstalk, suggesting that the autophagy-ferroptosis cross-pathway represents a potential therapeutic target for dry eye disease (168).

### 9.4 Ferroptosis and necroptosis

Ferroptosis and necroptosis can be triggered by the common stimulatory factor ROS and jointly participate in the process of oxidative stress-induced cell death (169). Tong et al. (170) demonstrated that treatment with the ferroptosis inducer (1S,3R)-RSL3 and the necroptosis inducer shikonin both triggered receptor-interacting protein kinase 3 (RIPK3) by lipid ROS accumulation and GPX4 inhibition, whereas necroptosis is accompanied by only a mild elevation of lipid ROS and is primarily marked by MLKL phosphorylation followed by translocation to the cell membrane, leading to increased membrane permeability. Further use of the RIPK1 inhibitor Nec-1 simultaneously suppressed the core components of both pathways, revealing their synergistic role in RPE injury. These findings suggest that targeting RIPK1 may represent a potential strategy for intervening in retinal degenerative diseases, such as AMD, and highlight the importance of combining multiple markers to distinguish between different types of cell death.

## 10 Innovations in treatment strategies based on ferroptosis

### 10.1 Therapeutic potential of ferroptosis inhibitors

Ferroptosis inhibitors exhibit the potential to suppress cell death, ameliorate tissue damage, and preserve visual function in multiple ophthalmic disease models through mechanisms such as scavenging lipid ROS, chelating iron ions, and regulating key signaling pathways (171). As classical ferroptosis inhibitors, the lipid peroxidation inhibitors Liproxstatin-1 (Lip-1) and Ferrostatin-1 (Fer-1) block the ferroptosis pathway by scavenging lipid ROS (172). Zhao et al. (173) demonstrated in an AMD model that Fer-1 and Lip-1 can mitigate cigarette smoke extract (CSE)-induced Fe^2+^ overload, ROS accumulation, and lipid peroxidation damage. These inhibitors restore GSH content and GPX4 activity, improve mitochondrial membrane potential decline and structural abnormalities such as mitochondrial shrinkage and reduced cristae, downregulate the expression of inflammatory cytokines, and reverse CSE-induced decreases in cell viability and damage to the retinal pigment epithelium-choroid complex. This study provides a potential therapeutic direction for inhibiting ferroptosis in smoking-related AMD. Sakamoto et al. (32) demonstrated that DFO and deferasirox protect retinal neurons by mitigating Fe^2+^ accumulation, reducing the expression of oxidative stress markers, and decreasing RGC loss. This provides a novel therapeutic strategy for excitotoxicity-related retinal diseases such as glaucoma.

Targeting the iron metabolism pathway: Nrf2 is not only a key factor involved in the oxidative stress response within cells but also participates in regulating the expression of key genes related to ferroptosis (174). Corilagin (COR) is a natural component found in various medicinal plants. It has been discovered that COR can increase the protein levels of Nrf2 and downstream antioxidant enzymes in the retinal tissues of diabetic mice, maintain the integrity of the retinal barrier structure, and reverse the increases in ROS and lipid peroxides induced by high glucose (175).

### 10.2 Smart nanoparticle systems

Nanoparticle drug delivery is a new type of drug administration method. It can effectively improve drug utilization and reduce the toxic and side effects of drugs. Currently, it is widely used in the medical field (176). Phenylboronic acid pinacol ester (PBA) was incorporated into Lycium barbarum polysaccharide (LBP) to synthesize PLBP nanoparticles. These nanoparticles scavenge intracellular ROS by activating the NRF2 pathway, inhibit ferroptosis in retinal ganglion cells (RGCs), and reduce microglial activation and inflammatory cytokine release via NF-κB pathway inhibition, offering a potential therapeutic strategy for retinal ischemia-related diseases (177).

Li et al. (178) conducted relevant research on using titanium dioxide nanoparticles (TiO_2_-NPs) as a sensitizer for sonodynamic therapy (SDT) to prevent posterior capsule opacification (PCO). During the research process, it was found that when TiO_2_-PLGA-coated intraocular lenses (IOLs) were combined with SDT, it could effectively reduce the proliferation of posterior capsular cells in a New Zealand white rabbit model of PCO and inhibit the accumulation of fibrotic substances. This combined application triggered the generation of ROS, resulting in the depletion of GSH and reducing the protein content of GPX4. The study pointed out that this therapy is likely to damage the proliferating HLECs by inducing intracellular ferroptosis, thereby achieving the purpose of preventing PCO.

### 10.3 Gene editing

Shen et al. (179) used transcriptome analysis to reveal mitochondrial shrinkage, lipid peroxidation, ROS accumulation, and increased PUFA levels in Cyp4v3 (homolog of human CYP4V2) knockout mouse RPE cells. Cao et al. (180) used bioinformatics to screen 10 ferroptosis key target genes mainly involved in oxidative stress and hypoxia response in PDR, providing new insights for clinical diagnosis and treatment through gene therapy.

### 10.4 Ocular delivery barriers and potential side effects

The blood–retinal barrier, composed of tight junctions in retinal vascular endothelial cells and the RPE, hinders the penetration of macromolecular drugs (such as iron chelators) into the posterior segment of the eye (181). A study has developed AN2728-Deferoxamine-Catechol Conjugate (ANCD) supramolecular nanoparticles self-assembled from DFO and crisaborole (AN). These nanoparticles simultaneously inhibit ferroptosis by chelating iron ions, scavenging free radicals, and inhibiting PDE4 enzyme to activate the cAMP/PKA pathway, thereby enhancing the survival rate of retinal ganglion cells (182). This approach significantly outperforms single-drug therapy and improves drug utilization efficiency. *In vivo* experiments showed no abnormalities in liver or kidney function, and local intraocular injection reduced systemic toxicity, indicating its translational medicine potential.

Ferroptosis has a promising future for the development of treatments for ocular diseases. It is expected to become a new therapeutic target for various refractory ocular diseases, such as age-related macular degeneration and glaucoma. By regulating key aspects to intervene in ferroptosis, the progression of these diseases can be delayed or even reversed. Meanwhile, it is conducive to the expansion of personalized medicine. Precise treatment plans can be formulated based on the detection of individual biomarkers. Moreover, it can bring new opportunities for drug research and development. New small-molecule drugs can be developed to enrich the treatment options.

## 11 Discussion

Since the discovery of ferroptosis in 2012 as an iron-dependent, non-apoptotic form of cell death, research into its mechanisms and its role in various diseases, including tumors, nerve injuries, inflammation, and infections, has progressed rapidly. The pathological roles of ferroptosis in different diseases are being increasingly elucidated. Notably, ophthalmologists have recognized the involvement of ferroptosis in the onset and progression of several retinal diseases, prompting further investigations into its key roles in major ocular conditions. However, the mechanisms of ferroptosis in retinal diseases remain under active study and require additional research and validation to inform the development of targeted therapeutic strategies. While some iron chelators have demonstrated promising therapeutic effects in animal models of retinal diseases, extensive clinical trials will be necessary to assess their feasibility for human treatment.

Based on the well-established molecular mechanisms of ferroptosis, current drug development presents diverse ideas. On the one hand, targeted intervention against iron metabolism-related proteins or receptors has become a popular direction. For example, the research and development of small molecule compounds that can regulate the expression or activity of iron transport proteins are being carried out, with the expectation of rebalancing the uptake and release of iron within cells. On the other hand, given the facilitating roles of inflammation and oxidative stress in the process of ferroptosis, anti-inflammatory and antioxidant drugs are also considered for alleviating ocular damage caused by ferroptosis. However, these strategies face numerous challenges during implementation. Due to the special physiological structure of the eye, the effective delivery of drugs and the specific targeting of different tissue cells within the eye remain issues that urgently need to be resolved. Meanwhile, while inhibiting ferroptosis, how to avoid adverse effects on normal iron metabolism and cellular functions is also a key factor that needs to be weighed in drug development.

Although this review has provided a comprehensive overview of the molecular mechanisms underlying ferroptosis in several ocular diseases, the mechanisms in certain rare ocular conditions remain insufficiently understood. Additionally, within the complex intracellular regulatory networks, many key molecular nodes and the details of their interactions are still poorly defined. This lack of clarity hinders the precision and effectiveness of targeted drug development. Most existing research has primarily focused on exploring fundamental molecular mechanisms and conducting validation in *in vitro* cell experiments and animal models. However, relatively few therapeutic agents have progressed to large-scale, high-quality clinical trials. As a result, there remains a substantial gap between laboratory findings and clinical application.

With a deeper understanding of the molecular processes involved in ferroptosis in ocular diseases, there is potential to develop a precision medicine approach tailored to individual patients, taking into account factors such as genetic background, iron metabolism status, and disease stage.
